# Gastroprotective effects of water extract of domesticated *Amauroderma rugosum* against several gastric ulcer models in rats

**DOI:** 10.1080/13880209.2022.2047210

**Published:** 2022-03-11

**Authors:** Yanzhen Mai, Siyuan Xu, Ru Shen, Bairu Feng, Hong He, Yifei Xu

**Affiliations:** a School of Pharmaceutical Sciences, Guangzhou University of Chinese Medicine, Guangzhou, China; b Huizhou Health Sciences Polytechnic, Huizhou, China; c Shenzhen Traditional Chinese Medicine Hospital, The Fourth Clinical Medical College of Guangzhou University of Chinese Medicine, Shenzhen, China

**Keywords:** Stomach, Traditional Chinese Medicine, anti-inflammatory, NF-κB/NLRP3

## Abstract

**Context:**

*Amauroderma rugosum* (Blume & T. Nees) Torrend (Ganodermataceae) is an edible mushroom with medicinal properties. However, the effects of *A. rugosum* on gastric ulcer remain unclear.

**Objective:**

To investigate the gastroprotective efficacy of water extract of *A. rugosum* (WEA) on gastric ulcer.

**Materials and methods:**

Sprague-Dawley rats were randomly grouped as control, model, lansoprazole and 200, 100 and 50 mg/kg of WEA. After pre-treatment for seven days, ethanol- and indomethacin-induced gastric ulcer models were established. The gastric ulcer and histopathology were investigated. Enzyme-linked immunosorbent assay (ELISA), quantitative polymerase chain reaction (Q-PCR) and Western blot assays were conducted to explore the potential anti-inflammatory effect and mechanism of WEA. Additionally, the pyloric ligation model was used to explore the influence of WEA on gastric acid and mucus.

**Results:**

Pre-treatment with WEA (200, 100 and 50 mg/kg) effectively reduced ulcerous area in both ethanol-induced (71%, 88% and 71%) and indomethacin-induced (77%, 65% and 86%) gastric ulcer model. The gastric levels of tumour necrosis factor-alpha (TNF-α) (34% and 50 mg/kg), interleukin-6 (IL-6) (32% and 100 mg/kg) and interleukin-1β (IL-1β) (36%, 45% and 41%) were reduced significantly (*p* < 0.05) by WEA. Serum nitric oxide was decreased significantly (*p* < 0.05) at 200 and 50 mg/kg and PGE2 concentration was increased remarkably (*p* < 0.05) at 100 mg/kg. Gene expression of inflammasome *Nlrp3*, and the nuclear translocation of nuclear factor-κB (NF-κB) P65 were significantly decreased by WEA pre-treatment. However, the pH of gastric acid and secretion of mucus did not show any significant change.

**Conclusions:**

The gastroprotective effect of WEA on gastric damage is attributed to anti-inflammation through the inhibition on NF-κB P65 nuclear migration and *Nlrp3* gene expression.

## Introduction

Gastric ulcer is a common and recurrent gastrointestinal disease affecting millions of people yearly (Greenwood-Van Meerveld et al. 2017). Gastric ulcer occurs due to various noxious factors, such as nonsteroidal anti-inflammatory drugs (NSAIDs), *Helicobacter pylori* infection, alcohol consumption, smoking, poor dietary habits and stress (Hunt et al. 2015). The imbalance between these noxious and protective factors (mucin, bicarbonate, prostaglandins, and so on) would trigger an interruption in the mucosal integrity, resulting in gastric mucosal lesions.

Numerous studies have indicated that inflammatory responses play an important role in the pathogenesis of acute gastric ulcer (Chien et al. 2015; Fu et al. 2018; Luo et al. 2018). The up-regulation of pro-inflammatory cytokines, such as tumour necrosis factor-alpha (TNF-α) and interleukins factors (IL‐1β and IL‐6), was observed in the acute phase of gastric ulceration (Hernández et al. 2014; Li Q et al. 2018). The nuclear translocation of nuclear factor-κB (NF-κB) P65 plays an essential role in the increment of TNF-α and interleukin-6 (IL-6), as well as NACHT, LRR and PYD domains containing protein 3 (NLRP3) inflammasome, which regulates the synthesis of interleukin-1β (IL-1β) (Doyle and O'Neill 2006; Zhen and Zhang 2019). Inhibition of gastric inflammatory responses is an effective therapy except for the control of gastric acid secretion by targeting type II histamine receptors and proton pumps for gastric ulcer treatment.

*Amauroderma rugosum* (Blume & T. Nees) Torrend (Ganodermataceae) is a traditional medicinal and edible mushroom. A notable characteristic is that the mushroom’s lower surface becomes red when it is touched (Chan et al. 2013), so it is also known as ‘blood Lingzhi’ in Chinese. *A. rugosum* lives in the tropics and subtropics, grows in humid and humus soil, attaches to the buried roots in hardwood forest (Seng et al. 2017). In recent years, the mushroom has been widely consumed because of both its nutritional value and wide range of medicinal properties. We have cultivated *A. rugosum* since 2015 in order to meet the ever-increasing market demand. *A. rugosum* has been used as a remedy to reduce inflammation, treat anuresis and upset stomach in China since ancient times (Lin et al. 2021). Modern pharmacological studies have shown that *A. rugosum* exhibited anti-inflammatory and antioxidant effects *in vitro* (Chan et al. 2015). However, pharmacological research of *A. rugosum* on gastrointestinal disease *in vivo* is rarely reported.

The present study investigated the gastroprotective efficacy of domesticated *A. rugosum* water extract (WEA) on rat gastric ulcer by using ethanol, indomethacin and pylorus ligation models. The gastroprotective and anti-inflammatory effects of WEA *in vivo* were demonstrated. Results provided a pharmacological basis for the use of *A. rugosum* in treating gastrointestinal diseases.

## Materials and methods

### Drugs and animals

Absolute ethanol was purchased from Tianjin Zhiyuan Reagent Co., Ltd. (Tianjin, China). Indomethacin was purchased from Meilunbio (Dalian, China). Lansoprazole was purchased from Renhe Pharmaceutical Co., Ltd. (Zhangshu, China). Male Sprague-Dawley rats (6–7 weeks old, 200–250 g) were obtained from Guangdong Medical Laboratory Animal Center (Foshan, China, SYXK (yue) 2018-0085). The rats were housed under specific pathogen-free conditions in a temperature-controlled room illuminated for 12 h every day and received humane care. This study was approved by the Animal Ethics Committee of Guangzhou University of Chinese Medicine (no. 20200826004). Prior to experimentation, all rats were fasted for 24 h and housed in cages with raised floors made of wide mesh to prevent coprophagy.

### Preparation of water extract of domesticated *Amauroderma rugosum*

Dried domesticated *A. rugosum* was purchased from Longmen Maling *Ganoderma lucidum* planting base (Huizhou, China) and identified by morphological, microscopic and gene sequencing (the internal transcribed spacer (ITS). Sequence analysis showed that the similarity between the sample (GenBank accession number: MK660145) and *A. rugosum* (GenBank accession number: MG021113.1) was 99%. The authenticated voucher specimen (voucher 20-07-02) was kept in Huizhou Health Sciences Polytechnic. Dried domesticated *A. rugosum* mycelia powder as above was extracted for 1 h in hot water (98 °C) in proportion (1:17, w/v) by heating reflux, thrice. The extracting solution was mixed, filtered, concentrated and lyophilized to obtain WEA. The powder of WEA was dissolved in appropriate distilled water to prepare the extract solution (1 mL equivalent to 1 g of *A. rugosum* mycelia powder) for test. The polysaccharide content was determined according to Chinese Pharmacopoeia (version 2020) based on the method of *Ganoderma lucidum* (Curtis: Fr.) P. Karst. (Ganodermataceae) polysaccharide quantification. The nucleosides were determined by high-performance liquid chromatography (HPLC). The total polysaccharides of WEA were analysed by anthrone-sulphuric acid colorimetry at 625 nm. The contents of four nucleosides, namely, including cytidine, uridine, guanosine and adenosine, were determined by HPLC.

InertSustain AQ-C18 (1.9 μm, 2.1 × 100 mm) column was used at 40 °C, and the flow rate was 0.3 mL/min. The injection volume was 1 μL, and the detection wavelength was set at 259 nm. The eluent was methanol (A)–water (B), and the gradient elution under HPLC conditions was as follows: 0–2 min, 0% A; 2–2.5 min, 0–2% A; 2.5–4 min, 2%A; 4–4.5 min, 2–10% A; 4.5–8.5 min, 10%A; 8.5–9.0 min, 10–30% A; 9.0–10.5 min, 30–90% A.

### Groups and drug administration

Rats were randomly divided into six groups, namely, the blank control (*n* = 6), the gastric ulcer model (*n* = 8), lansoprazole (*n* = 6), H-WEA (*n* = 6), M-WEA (*n* = 6) and L-WEA (*n* = 6). The number of rats were adjusted to six in pyloric ligation assay. The details of drug administration were as follows. The blank control group and gastric ulcer model rats were treated with distilled water (1 mL/100 g, p.o.) for seven consecutive days. The rats in the lansoprazole were treated with 30 mg/kg lansoprazole. An acute toxicity assay of *A. rugosum* indicated that oral administration of a single dose of the powder (2000 mg/kg) had no adverse effect (Fung et al. 2017). According to the results of our pre-experiment, the H-WEA, M-WEA and L-WEA groups were treated with 200, 100 and 50 mg/kg WEA (p.o.) for seven consecutive days, respectively. All rats were fasted for 24 h with free access to drinking water in the last day.

### Ethanol-induced gastric ulcer model assay

One hour after the last treatment, rats (except for those in the blank control group) received an oral dose of 1 mL of absolute ethanol. After 1 h, all rats were anaesthetized with isoflurane, blood was collected from the abdominal aorta and serum was separated after centrifugation. Stomachs were harvested. The stomachs were then opened from the greater curvature and rinsed with cold saline to remove the gastric contents and blood clots. The flattened stomach samples were photographed, and the ulcerated area (mm^2^) was measured using Image J software (developed by the National Institutes of Health, Bethesda, MD). The stomach was cut into three parts, as follows. One was fixed in 10% paraformaldehyde for pathological section observation. One was frozen for the detection of inflammatory response. One was dipped in RNAwait (Meilunbio, Dalian, China) solution to measure gene expression.

### Indomethacin-induced gastric ulcer model assay

Thirty minutes after the last treatment, rats (except for the rats in the blank control group) were given an oral dose of 100 mg/kg of indomethacin. After 5 h, all rats were euthanized with carbon dioxide, and the subsequent procedures were performed in accordance with the ethanol-induced gastric ulcer protocol.

### Pyloric ligation model assay

After the oral administration of the last drug or distilled water for 1 h, the rats (except for the rats in the control) were subjected to longitudinal incisions slightly below the xiphoid apophysis to place a pyloric ligature (Moawad et al. 2019). After 4 h, the rats were sacrificed, the abdomen was opened, and another ligature was placed around the oesophagus close to the diaphragm. The stomach was opened along the greater curvature, and the gastric contents were collected and centrifuged for 10 min at 5000×*g*. The pH of gastric juice was measured by pH meter. The glandular portion of the stomach was weighed and immersed for 2 h in alcian blue solution (Sigma-Aldrich, St. Louis, MO) with vortexing every 10 min for the mucus quantification procedure. The absorbance was measured in a spectrophotometer (Multiskan GO, Thermo Fisher Scientific, Waltham, MA) at a wavelength of 585 nm, and the results were expressed as μg Alcian Blue/g tissue (Rafatullah et al. 1990).

### Inflammatory cytokines and nitrogen monoxide (NO) evaluation

Tissues were homogenized in normal saline. Supernatant was harvested after centrifuging at 10,000×*g* for 15 min at 4 °C. Tumour necrosis factor-α, IL-1β and IL-6 were measured by commercial enzyme-linked immunosorbent assay (ELISA) kits from 4 A Biotech (Beijing, China). Prostaglandin E2 (PGE2) was determined by commercial kit from ANRC (Tianjin, China). NO was measured by commercial kits from Beyotime Biotechnology (Shanghai, China).

### Haematoxylin and eosin staining assay

Stomach tissues were fixed in formalin, paraffin-embedded, sectioned and stained with haematoxylin and eosin. The histological examination of gastric tissue was performed according to the methods presented previously (Luo et al. 2018).

### Real-time quantitative polymerase chain reaction (q-PCR)

Total RNA was isolated by homogenizing the tissues in RNApure Tissue & Cell Kit (Beijing, China), and single standard cDNA was synthesized by using Tiangen cDNA kit (Beijing, China). Quantitative real-time PCR was performed with Thermo Scientific QuanStudio™ 5 Real-Time PCR instrument (Waltham, MA). Primer sequences are listed in Table 1.

**Table 1.** Primer sequences for real-time PCR assays.

Gene	Primer (5′–3′)
*Gapdh*	f GGCACAGTCAAGGCTGAGAATG
	r ATGGTGGTGAAGACGCCAGTA
*Cox2*	f TGAACACGGACTTGCTCACTTTG
	r AGGCCTTTGCCACTGCTTGTA
*Nlrp3*	f CTGAAGCATCTGCTCTGCAACC
	r AACCAATGCGAGATCCTGACAAC
*Caspase-1*	f ACTCGTACACGTCTTGCCCTCA
	r CTGGGCAGGCAGCAAATTC
*Asc*	f TTGCTGGATGCTCTGTATGG
	r CAGGTCTGTCACCAAGTAGG

Sequences: 5′–3′. Forward primers are designated by f and reverse primers by r.

### Western blot

Nuclear and cytoplasmic proteins were extracted using NE-PER™ Nuclear and Cytoplasmic Extraction Reagents from Thermo Fisher (Waltham, MA). Protein extracts were fractionated by sodium dodecyl sulphate polyacrylamide gel electrophoresis and transferred to polyvinylidene difluoride membranes. The membranes were then blocked with 5% non-fat milk in Tris-buffered saline with Tween-20 for 2 h at room temperature and incubated with anti-β-actin (SAB, MD, USA, 1:5000), anti-Histone H3 (SAB, 1:5000) and anti-NF-κB P65 (SAB, 1:1000) at 4 °C overnight. They were rinsed thrice with TBST and incubated with respective secondary antibodies for 2 h at room temperature. Protein bands were visualized with Millipore Immobilon Western Chemiluminescent HRP Substrate (Boston, MA) and captured using the Bio-Rad ChemiDoc™ Imaging System imaging system (Shanghai, China).

### Data analysis

All results were expressed as means ± standard error. Data from more than two groups were analysed by one-way ANOVA followed by LSD or Dunnett’s test for multiple comparisons. Student’s *t-*test was performed to identify differences between two groups. *p* < 0.05 was considered significant.

## Results

### Quantification of four nucleosides and total polysaccharides in WEA

**Figure 1.**
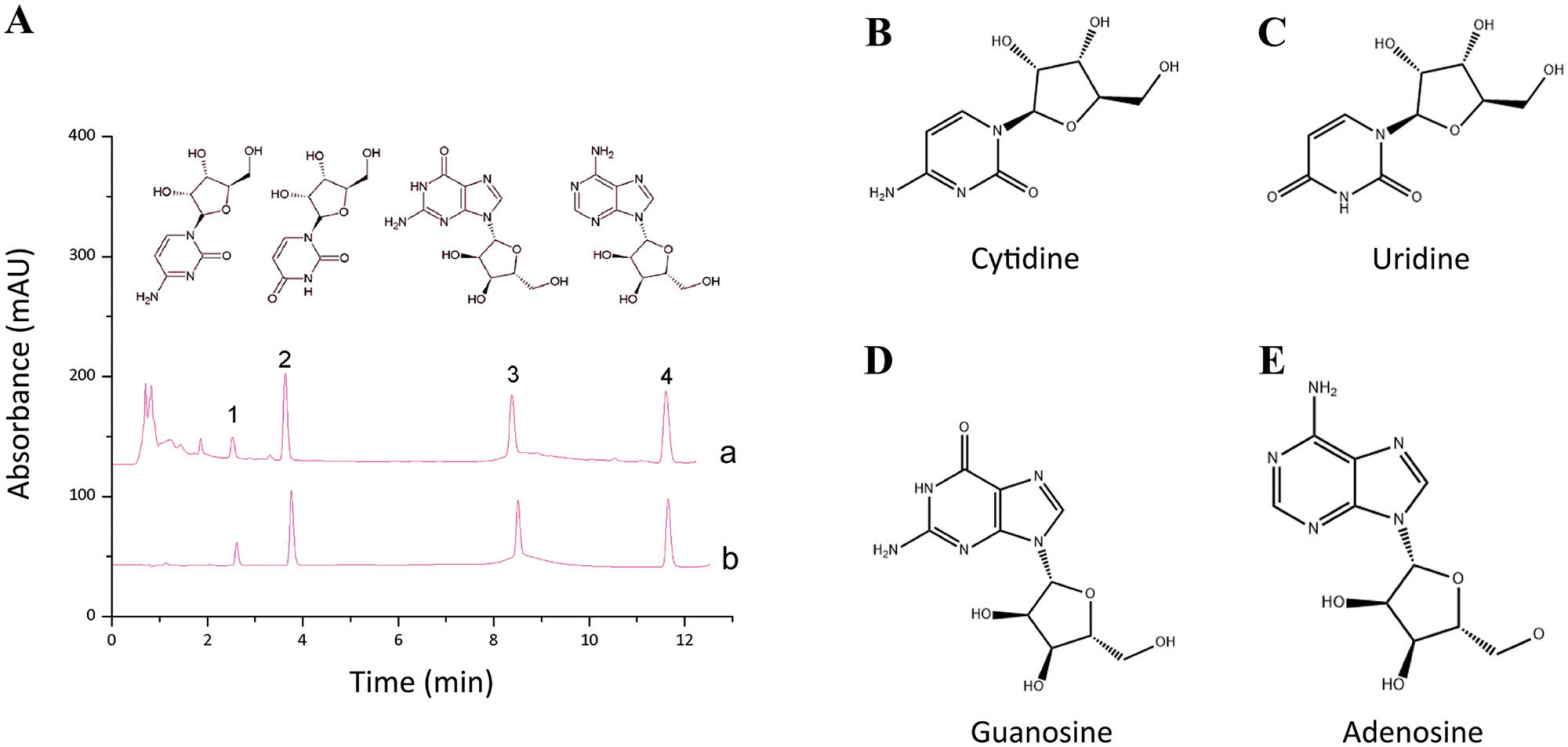
Specific components of WEA determined by HPLC. (A) High-performance liquid chromatography result of WEA (a: test solution; b: reference solution); (B) cytidine; (C) uridine; (D) guanosine; (E) adenosine.

The yield of WEA was 6.13%, RSD was 7.52%. HPLC and visible spectrophotometry analysis were conducted to quantify the four nucleosides and total polysaccharides in the WEA. The cytidine, uridine, vernine and adenosine were 0.2465, 0.6301, 0.4064 and 0.4989 mg/mL, respectively. The total polysaccharides were 7.2 mg/mL (Table 2, Figure 1).

**Table 2.** Composition of WEA analysed by HPLC and visible spectrophotometry (*n* = 3).

Nucleosides and polysaccharides	Concentration (mg/mL)	RSD (%)
Cytidine	0.2465	3.87
Uridine	0.6301	3.00
Vernine	0.4064	4.40
Adenosine	0.4989	3.99
Total nucleosides	1.7819	1.53
Total polysaccharides	7.20	2.92

### WEA exhibited gastroprotective effect in ethanol-induced gastric ulcer

**Figure 2.**
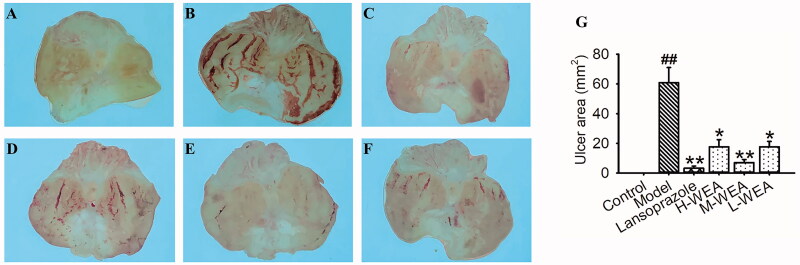
Effects of WEA on the appearance of the gastric mucosa in ethanol-induced gastric lesions in rats. (A) Intact gastric mucosa of the normal control; (B) gastric mucosa of ethanol-induced gastric lesions; (C) gastric mucosa after lansoprazole administration; (D–F) gastric mucosa after the H-WEA (200 mg/kg), M-WEA (100 mg/kg) and L-WEA (50 mg/kg) administration; (G) gastric ulcer area (mm^2^) of rat stomach induced by ethanol after pre-treatment with lansoprazole and WEA at different dosages. Values are means ± SEMs (*n* = 8 for model group, *n* = 6 for other groups). ^##^*p*< 0.01 vs. the control group; *p< 0.05, **p< 0.01 vs. the model group.

After receiving absolute ethanol 1 h, ethanol-induced gastric ulcer model rats exhibited severe damage in the ulcerated area (60.84 ± 10.16 mm^2^) (Figure 2(B,G)). This severe ulcerative injury was reversed by lansoprazole pre-treatment, and the ulcerated area was reduced significantly with lansoprazole pre-treatment (3.22 ± 1.43 mm^2^, reduced 95%) (Figure 2(C,G)). Meanwhile, all three dosages of WEA pre-treatment attenuated the ulcerative damage (17.68 ± 4.74, 7.07 ± 2.01 and 17.70 ± 3.57 mm^2^; reduced 71%, 88% and 71%) (Figure 2(D–G)). Reduction of ulcerated area was observed in WEA pre-treatment group (Figure 2(G)).

### WEA could protect rat from indomethacin-induced gastric ulcer

**Figure 3.**
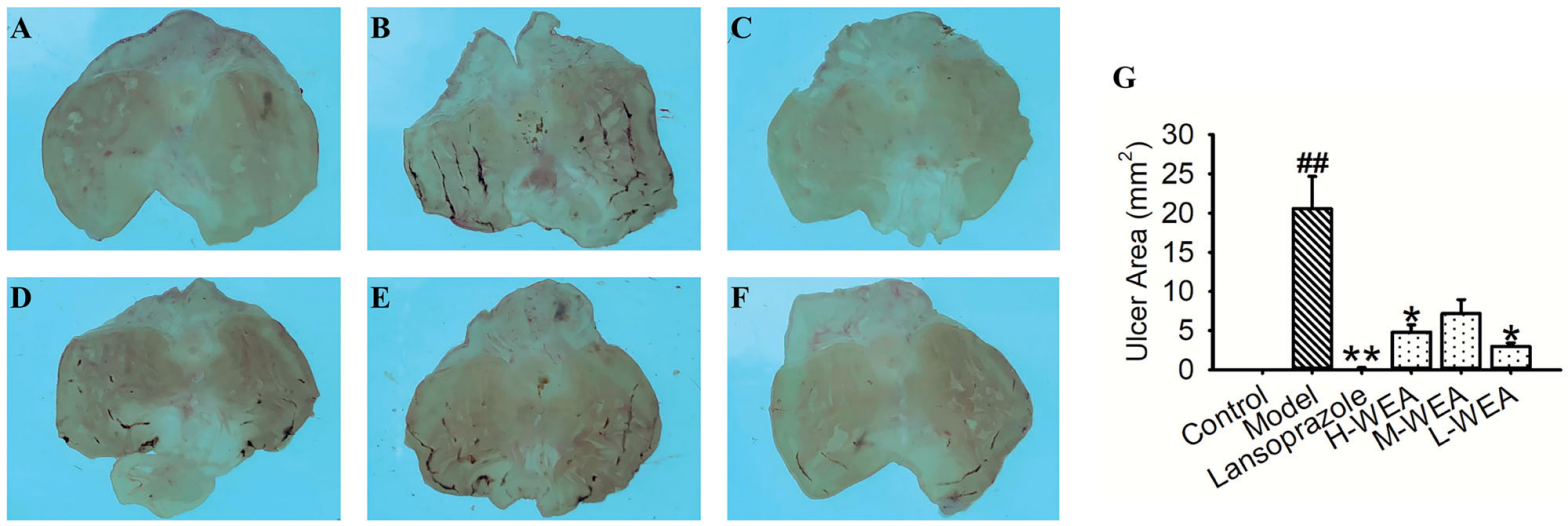
Effects of WEA on the appearance of the gastric mucosa in indomethacin-induced gastric lesions in rats. (A) Intact gastric mucosa of normal control; (B) gastric mucosa of indomethacin-induced gastric lesions; (C) gastric mucosa after lansoprazole administration; (D–F) gastric mucosa after H-WEA (200 mg/kg), M-WEA (100 mg/kg) and L-WEA (50 mg/kg) administration; (G) gastric ulcer area (mm^2^) of rat stomach induced by indomethacin after pre-treatment with lansoprazole and WEA at different dosages. Values are means ± SEMs (*n* = 8 for model group, *n* = 6 for other groups). ^##^*p*< 0.01 vs. control group; *p< 0.05, **p< 0.01 vs. model group.

After intervention with indomethacin at 100 mg/kg, rats showed mild mucosal injury in the ulcerated area (20.58 ± 4.12 mm^2^) (Figure 3(B,G)). Consistent with ethanol-induced gastric ulcer, mucosal injury and ulcerated area were also alleviated with lansoprazole pre-treatment (0.22 ± 0.05 mm^2^, reduced 99%) (Figure 3(C,G)). H-WEA and L-WEA pre-treatments ameliorated the indomethacin-induced mucosal injury, and the ulcerated area decreased after WEA pre-treatment (4.80 ± 0.95, 7.17 ± 1.80 and 2.97 ± 0.45 mm^2^; reduced 77%, 65% and 86%) (Figure 3(D–G)).

### Histological evaluations of ethanol-induced gastric ulcer

**Figure 4.**
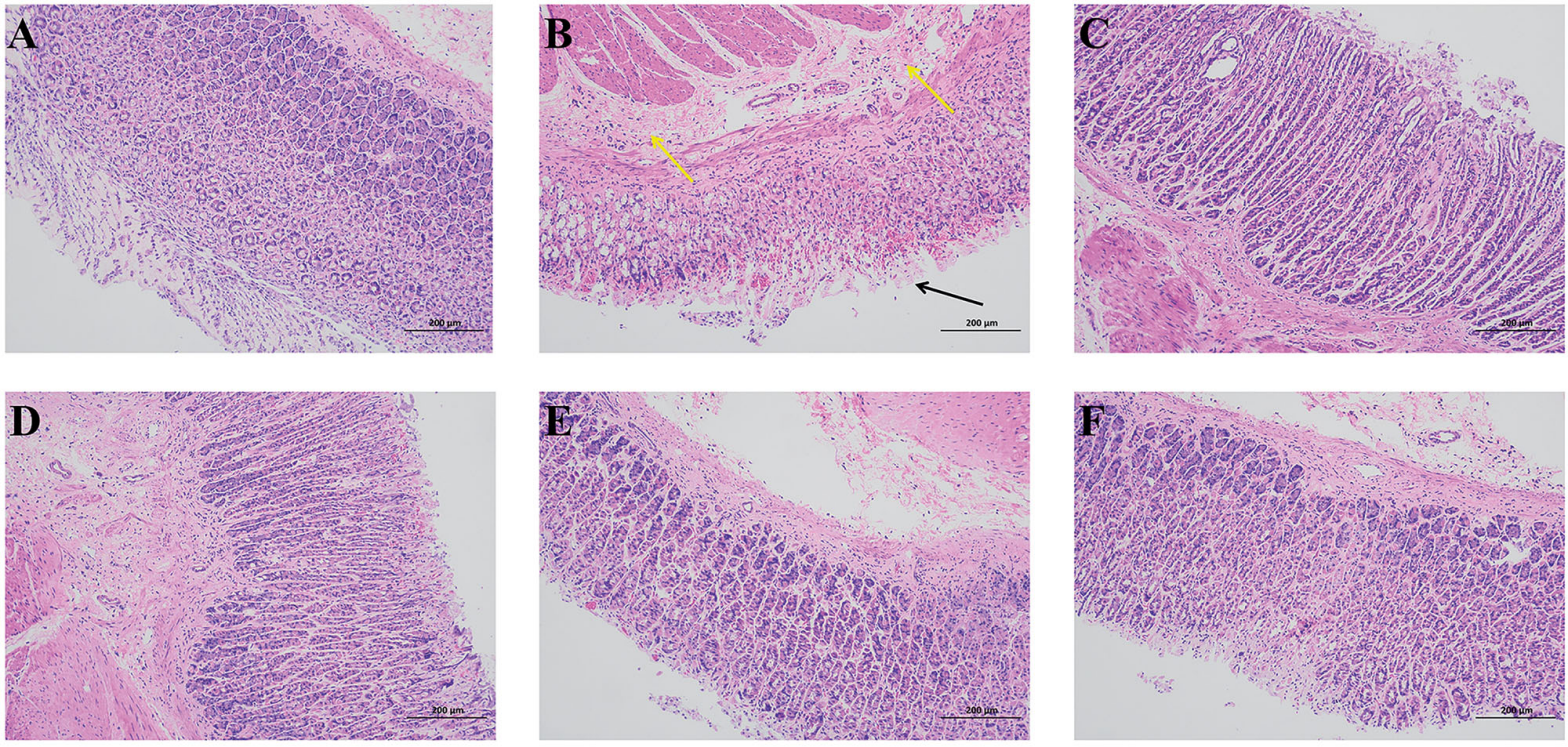
Effects of WEA on histological evaluation in ethanol-induced gastric lesions in rats (HE staining; magnification ×100). (A) Intact gastric epithelium with organized glandular structure and normal submucosa; (B) ethanol-induced ulcer (black arrow: necrocytosis of gastric epithelium; yellow arrow: oedema of submucosa); (C) lansoprazole administration; (D–F) H-WEA (200 mg/kg), M-WEA (100 mg/kg) and L-WEA (50 mg/kg) administration.

In the histological analysis, rats exhibited extensive erosion in the mucosal layer (Figure 4(B)). Necrocytosis of gastric epithelium and oedema of submucosa with inflammatory infiltration were observed (Figure 4(B)). Lansoprazole pre-treatment could prevent the rat from ethanol-induced erosion in the mucosal layer; necrocytosis of gastric epithelium and oedema of submucosa were also diminished (Figure 4(C)). The necrocytosis of gastric epithelium and oedema of submucosa, as well as the erosion of the mucosal layer, were reversed by 200 mg/kg WEA (H-WEA) pre-treatment. (Figure 4(D)). Rats pre-treated with 100 mg/kg WEA (M-WEA) could also improve the histological exhibition in the gastric epithelium and submucosa (Figure 4(E)). In the 50 mg/kg WEA (L-WEA) pre-treatment group, slight exfoliation of epithelial cells and oedema of submucosa were observed (Figure 4(E)). These results showed that WEA pre-treatment can prevent ethanol-induced damage on the gastric area.

### Effects of WEA on inflammatory cytokines, NO and PGE2 secretion in ethanol-induced gastric ulcer

**Figure 5.**
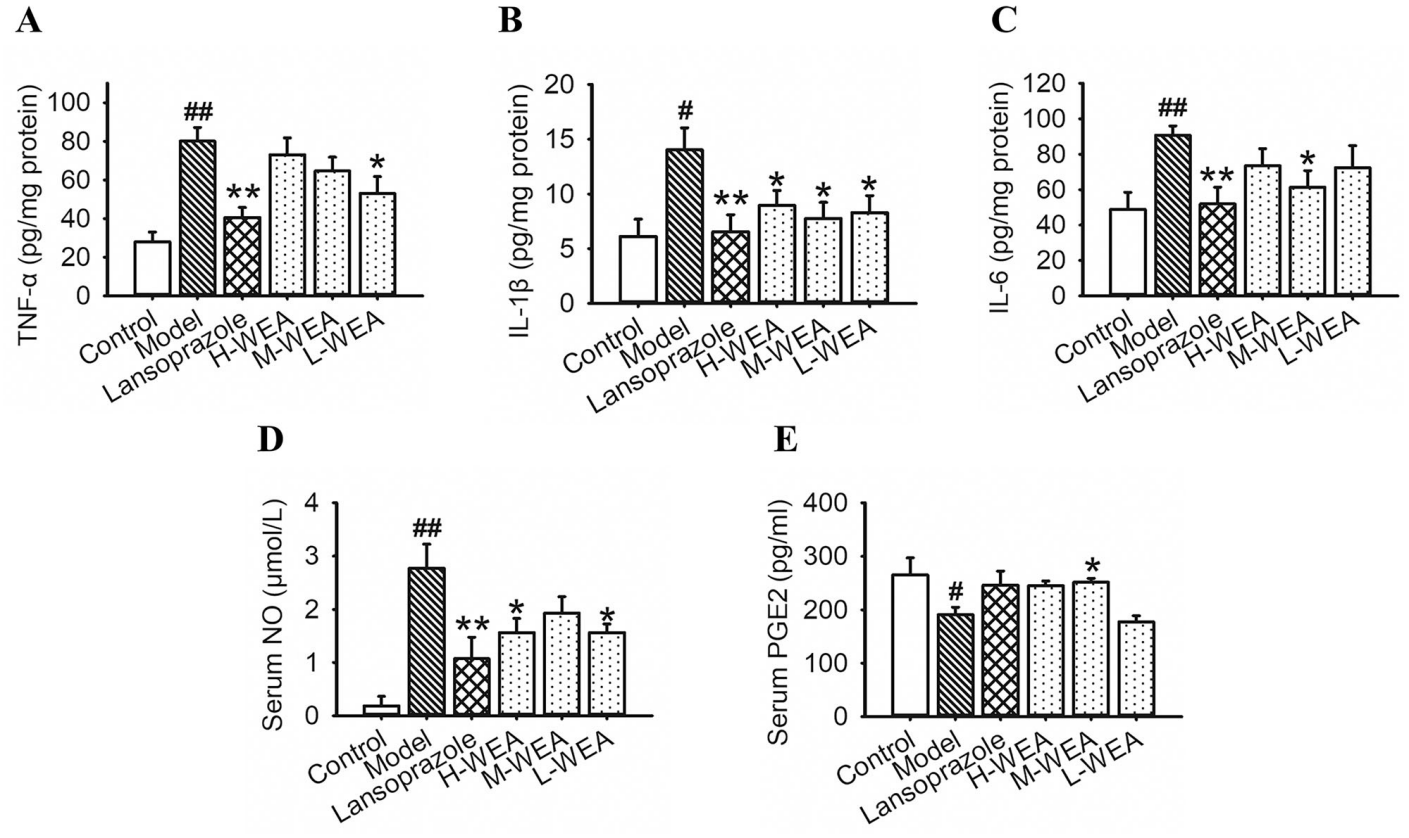
Effects of WEA on inflammatory cytokines, NO and PGE2 secretion in ethanol-induced gastric lesions in rats. (A) Gastric TNF-α; (B) gastric IL-1β; (C) gastric IL-6; (D) serum NO; (E) serum PGE2. Values are means ± SEMs (*n* = 8 for model group, *n* = 6 for other groups). ^#^*p*< 0.05, ^##^*p*< 0.01 vs. the control group; *p< 0.05, **p< 0.01 vs. the model group.

Based on the above findings that WEA attenuated the ethanol-induced gastric ulcer and improved the histological morphology, we assumed that the secretion of inflammatory cytokines was suppressed by WEA pre-treatment. A significant increase in TNF-α, IL-1β and IL-6 secretions in stomach tissues was observed after ethanol intervention, which indicated that severe inflammatory infiltration occurred in the stomach (80.12 ± 7.10, 14.03 ± 2.01 and 90.73 ± 5.20 pg/mg) (Figure 5(A–C)**)**. Lansoprazole pre-treatment significantly reduced the levels of these inflammatory cytokine (40.68 ± 5.19, 6.52 ± 1.59 and 51.92 ± 9.48 pg/mg; reduced 49%, 54% and 43%) (Figure 5(A–C)). L-WEA pre-treatment significantly decreased the gastric level of TNF-α (53.03 ± 8.78 pg/mg, reduced 34%) (Figure 5(A)). Moreover, all three dosages of WEA exhibited significant effects on the lowering of IL-1β level (8.95 ± 1.37, 7.77 ± 1.45 and 8.29 ± 1.56 pg/mg; reduced 36%, 45% and 41%) (Figure 5(B)). M-WEA could significantly reduce IL-6 level (61.40 ± 9.38 pg/mg, reduced 32%) (Figure 5(C)). Consistent with the results on inflammatory cytokines, serum NO also increased after ethanol administration (2.77 ± 0.45 µmol/L) (Figure 5(D)). However, this effect was neutralized by both H-WEA and L-WEA pre-treatments (1.56 ± 0.27, 1.57 ± 0.16 µmol/L; reduced 44%, 43%) (Figure 5(D)). Serum PGE2 decreased with ethanol administration (190.83 ± 14.15 pg/mL) (Figure 5(E)), but this result was reversed by M-WEA pre-treatment (251.55 ± 7.18 pg/mL, increased 31.82%) (Figure 5(E)).

### Effects of WEA on gene expression in gastric ulcer

**Figure 6.**
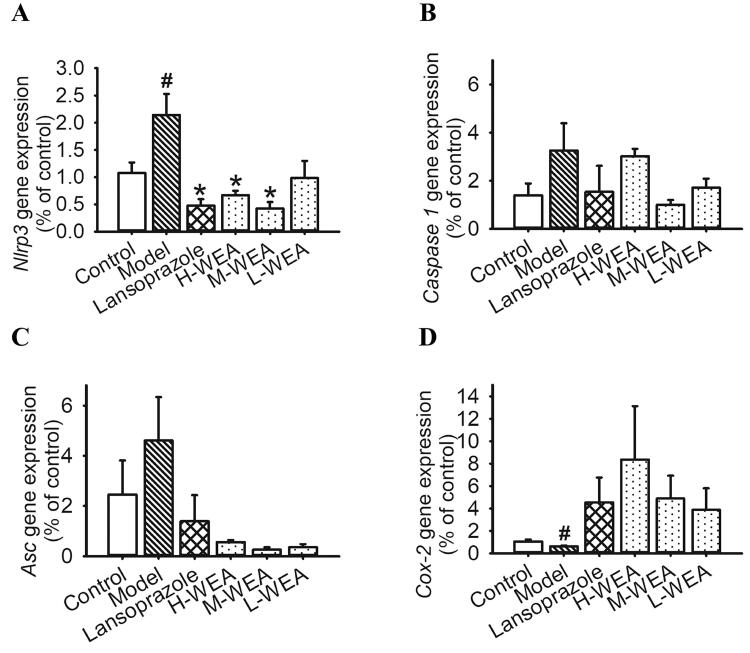
Effects of WEA on gastric *Nlrp3*, *Caspase-1*, *Asc* and *Cox-2* mRNA expression in ethanol- and indomethacin-induced gastric lesions in rats. (A–C) *Nlrp3*, *Caspase-1* and *Asc* relative gene expression in rat stomach induced by ethanol after pre-treatment with lansoprazole and WEA at different dosages (*n* = 8 for the model group, *n* = 6 for the other groups); (D) *Cox-2* gene expression in the rat stomach induced by indomethacin after pre-treatment with lansoprazole and WEA at different dosages (*n* = 5 per group). Values are means ± SEMs. ^#^*p*< 0.05 vs. the control group; *p< 0.05 vs. the model group.

After investigating the anti-inflammatory effects of WEA on rats, we determined the inflammatory relative gene expression in ethanol- and indomethacin-induced gastric ulcer rats after WEA intervention. In rats with ethanol-induced ulcer, a critical pro-inflammatory mediator *Nlrp3* was over-expressed in the stomach after ethanol administration (Figure 6(A)). Lansoprazole, H-WEA and M-WEA significantly suppressed gene expression of *Nlrp3* in the gastric tissue (Figure 6(A)). In the indomethacin-induced ulcer rats, the gene expression of *Cox-2* was inhibited (Figure 6(A)). Although WEA pre-treatment enhanced *Cox-2* expression, no significant difference was found (Figure 6(A)).

### WEA inhibited the nuclear migration of NF-κB P65 in ethanol-induced gastric ulcer

**Figure 7.**
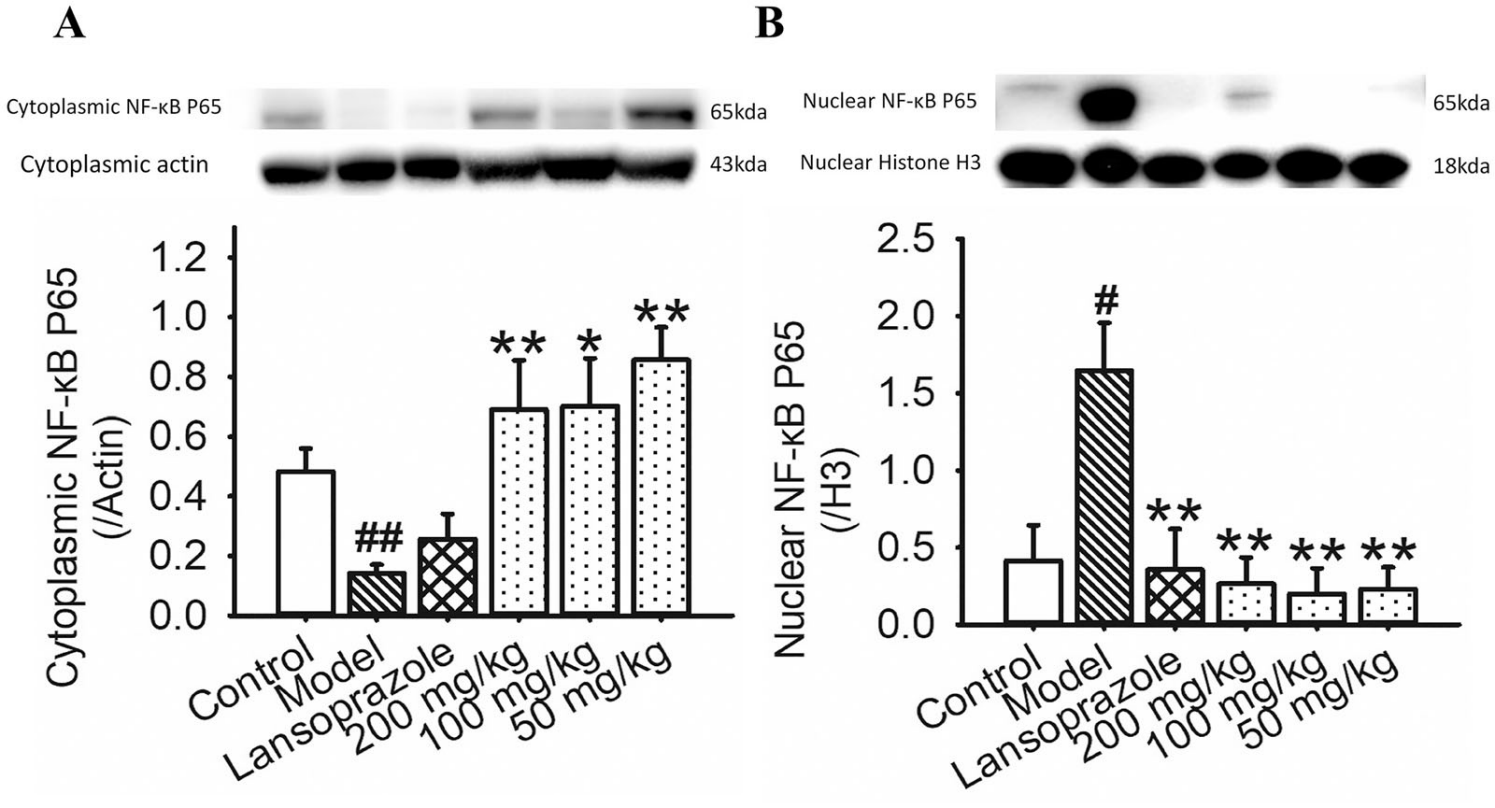
Effects of WEA on the expression of NF-κB P65 in ethanol-induced gastric lesions in rats. (A) Visualization and quantification of cytoplasmic NF-κB P65; (B) visualization and quantification of the expression of nuclear NF-κB P65. Values are means ± SEMs (*n* = 4 per group). ^#^*p*< 0.05, ^##^*p*< 0.01 vs. the control group; *p< 0.05, **p< 0.01 vs. the model group.

We estimated the levels of cytoplasmic/nuclear NF-κB P65 to further explore the anti-inflammatory mechanism of WEA in gastric ulcer treatment. The level of gastric nuclear NF-κB P65 was significantly increased after ethanol intervention, and the level of cytoplasmic NF-κB P65 decreased (Figure 7). These data suggested that the cytoplasm–nuclear transfection was activated by ethanol stimulation, which was a critical target for acute inflammation activation. Lansoprazole pre-treatment could suppress the nuclear migration of NF-κB P65 significantly (Figure 7). Compared with lansoprazole treatment, WEA pre-treatment inhibited the increase of nuclear NF-κB P65 and recovered the cytoplasmic level of NF-κB P65 (Figure 7). Thus, WEA pre-treatment can prevent the ethanol-induced cytoplasm-nuclear NF-κB P65 transfection.

### Effects of WEA on pyloric ligation rats

After rats were subjected to pyloric ligation for 4 h, the retention of gastric juice in the stomach was observed (Table 3). We compared the gastric mucus and pH of gastric juice after pyloric ligation with WEA administration. However, the three dosages of WEA pre-treatment failed to normalize the pH of gastric juice (Table 3). Lansoprazole pre-treatment even up-regulated the pH of gastric juice (Table 3). On the contrary, gastric mucus was diminished after pyloric ligation (Table 3). However, both lansoprazole and WEA administration had no significant effects on this parameter (Table 3).

**Table 3.** Effects of WEA on the pH and mucus in rats with pyloric ligation.

Groups	pH	Mucus (μg Alcian blue/g tissue)
Control	–	1809.93 ± 135.32
Model	2.55 ± 0.10	1065.59 ± 64.47^##^
Lansoprazole	6.53 ± 0.25**	896.34 ± 79.01
200 mg/kg	2.30 ± 0.05	1238.84 ± 164.63
100 mg/kg	2.52 ± 0.06	1344.66 ± 112.10
50 mg/kg	2.43 ± 0.08	1211.00 ± 144.85

Values are means ± SEMs, *n* = 6 per group.

^##^*p*< 0.01 vs. control group.

**p< 0.01 vs. model group.

## Discussion

Gastric ulcer is among the most prevalent gastrointestinal diseases in the world. Many studies revealed the protective effects of edible mushrooms and nature products against gastric mucosa diseases (Wang M et al. 2015; Khémiri and Bitri 2019; Wang L et al. 2020). *A. rugosum*, an edible and medicinal mushroom, was demonstrated to have anti-inflammation and antioxidant effects in recent years (Chan et al. 2013, 2015; Li J et al. 2021). However, the effects of *A. rugosum* on gastric mucosa damage is still unclear. In this study, several gastric ulcerative animal models were established to investigate the gastroprotective efficacy of WEA, and results indicated that WEA exhibited anti-inflammatory effects on various aspects.

Oral administration of absolute ethanol is a well-recognized method for inducing gastric injury in animal models (Kwon et al. 2019). In this model, rats subjected to ethanol showed severe gastric lesions, most of which involved extensive erosion in the mucosal layer and necrocytosis of the gastric epithelium. Meanwhile, ethanol administration led to elevated serum NO. NO, a free radical molecule, plays different roles under physiological and pathophysiological conditions. At physiological concentration, NO plays a role against gastric mucosal damage (Elliott and Wallace 1998). Recent research indicated that ethanol administration could enhance inducible nitric oxide synthase (iNOS) expression and inhibit the expression of endothelial nitric oxide synthase (eNOS), leading to NO overload and subsequent cytotoxic effects and free radical generation (Bagyánszki et al. 2011; Amirshahrokhi and Khalili 2015). WEA pre-treatment significantly ameliorated the ethanol-induced gastric mucosal lesion and reversed the elevated serum NO, which suggested that WEA has potential effects on the restoration of antioxidants in ethanol-induced gastric ulceration. In addition, ethanol administration decreased the serum PGE2, which is an endogenous gastroprotective mediator that promotes gastric ulcer healing by improving the secretion of bicarbonate and mucus. PGE2 can also increase gastric mucosal blood flow and protect against reactive oxygen species (ROS) in the stomach (Amirshahrokhi and Khalili 2017; Cheng et al. 2017). WEA pre-treatment significantly increased the concentration of serum PGE2.

Inflammatory cytokines play pivotal roles in gastric mucosal damage (Chien et al. 2015; Fu et al. 2018; Luo et al. 2018). In this research, ethanol administration stimulated inflammatory infiltration. The increase of several inflammatory cytokines, such as TNF-α, IL-6 and IL-1β, in gastric tissue was observed. These cytokines could deteriorate oxidative stress by accelerating mitochondrial ROS generation and cytotoxicity (Rozza et al. 2014; Rajamanickam et al. 2020). Moreover, TNF-α can induce the immune response to generate cytotoxic metabolites, suppress gastric microcirculation and delay the recovery (Verma and Kumar 2016). Many studies have indicated that the levels of IL-6 and IL-1β in gastric tissue are correlated with the severity of gastric ulceration (Li WF et al. 2014). In the present study, WEA pre-treatment significantly decreased the levels of gastric TNF-α, IL-6 and IL-1β, which indicated that WEA possessed anti-inflammatory effects on ethanol-induced gastric ulceration. We aimed to investigate the mechanism underlying the gastroprotective effects of WEA. Interestingly, another critical pro-inflammatory mediator involved in gastric ulceration, NLRP3, was over-expressed in gastric tissue. NLRP3 inflammasome, assembled by NLRP3, ASC and pro-CASPASE-1, would be activated under gastric ulcer conditions, resulting in the proteolysis of pro-CASPASE-1 into cle-CASPASE-1 and leading to the release of functional IL-1β (Swanson et al. 2019). The activation of NLRP3 inflammasome can be triggered by NF-κB pathway and result in the increase of IL-1β and TNF-α secretion (Chi et al. 2015; Ma et al. 2018), as well as the increase in nuclear migration of NF-κB P65. In agreement with the effects of inflammatory cytokines, WEA pre-treatment also suppressed the nuclear migration of NF-κB P65 and inactivated the NLRP3 inflammasome gene expression. We suggested that WEA could inhibit gastric inflammation by suppressing the NF-κB/NLRP3 pathway and attenuating the inflammatory cytokine-associated oxidative stress.

Indomethacin, an NSAID, was used to induce a gastric ulcer model, which is used to confirm the gastroprotective effects of WEA in different ulcerous animal models. Indomethacin is a non-selective inhibitor of both COX-1 and COX-2 and can cause damage in the stomach (Zheng et al. 2014; Stachowicz 2020). WEA pre-treatment could also attenuate indomethacin-induced gastric ulceration. Although WEA had no significant effect on the gastric *Cox-2* gene expression, the gastroprotective effects and the reliable anti-inflammation effect of WEA were still robust. A pylorus ligation gastric ulcer model was performed to investigate the different gastroprotective effects of WEA on gastric ulceration and the underlying mechanism. In this model, the ligation of the pyloric end leads to the accumulation of gastric acid, which is another pathological feature of gastric ulceration. The decrement of mucus secretion results in the erosion by gastric acid, which is the pivotal factor for the start of ulceration (Adeniyi et al. 2018). Therefore, gastric acid accumulated in the stomach, and the mucus secretion was reduced after pylorus ligation, thereby resulting in gastric mucosa damage. WEA pre-treatment had no significant effects on the pH of gastric acid and the secretion of mucus. Based on the above evidence, we suggested that the therapeutic effects of WEA on gastric ulcer are independent of hydrochloric acid production.

WEA can alleviate gastric ulcer through the inhibition of nuclear migration of the critical inflammatory factor NF-κB P65 and the inactivation of the NF-κB/NLRP3 pathway. Although the reliable anti-inflammation effect of WEA may contribute to the improvement of indomethacin-induced gastric ulcer, the specific mechanism of WEA in non-steroid anti-inflammatory drug-induced ulcer may need further investigation. *A. rugosum* has potential to be further developed into a promising candidate for the treatment of gastric ulcer.
